# Case Report: Lung Ultrasound in Critically Ill Neonates With Lung Diseases: Experience From Several Typical Cases

**DOI:** 10.3389/fped.2022.846279

**Published:** 2022-05-19

**Authors:** Biying Deng, Fengdan Xu, Jinfeng Li, Minling Mai, Qin Chen, Jinfeng Liao, Xiaoguang He, Ning Li

**Affiliations:** Department of Neonatal Intensive Care Unit, Guangdong Medical University Affiliated Dongguan Children’s Hospital, Dongguan, China

**Keywords:** lung ultrasound, neonate, lung diseases, pulmonary edema, pneumothorax, lung recruitment, consolidation

## Abstract

Lung ultrasound (LUS) can be used to diagnose various neonatal lung diseases. It more sensitively diagnoses pulmonary edema, pneumothorax, pulmonary consolidation, and atelectasis than traditional X-ray and quickly determines the cause of dyspnea. As a component of severe ultrasound, LUS enables rapid bedside visualization of lung diseases and plays a major role in guiding the differential diagnosis of disease, ventilator treatment, and lung recruitment. This study introduced the application of LUS in the diagnosis and treatment of critically ill neonates with lung diseases.

## Introduction

Respiratory diseases are the leading causes of neonatal death. The common respiratory diseases include meconium aspiration syndrome (MAS), respiratory distress syndrome (RDS), pneumothorax (PTX), pneumonia (PN), and transient tachypnea of the newborn (TTN). Due to the physiological characteristics of the respiratory tract and poor alveolar compensation capacity of the neonates, mechanical ventilation respiratory support is needed in most cases. However, it may develop into critical ill respiratory disease. The treatment of such a disease has always been challenging and the focus of neonatal medicine; thus, the accurate and rapid examination is the key to successful treatment. In the last decade, with the development of technology, lung ultrasound (LUS) has been successfully applied in the clinical diagnosis of various neonatal lung diseases, thereby opening a new era of green diagnosis of lung diseases (1–3). The advantages of ultrasound are no longer limited to diagnosis. Ultrasound also plays a major role in guiding the clinical treatment of critically ill respiratory cases.

## Identification of “White Lung” in X-ray: Respiratory Distress Syndrome or Transient Tachypnea of the Newborn?

“White lung,” the most critical condition of the respiratory system, refers to the extensive exudation and consolidation of both lungs caused by various reasons. Chest X-ray displayed that cardiac margin and diaphragmatic surface are not clear, and the lung development is white. The most common neonatal lung diseases with white lung on chest X-rays are severe respiratory distress syndrome (RDS) and severe TTN. Clinically, these two lung diseases have similar history and symptoms, but their pathophysiological changes and treatment have some variations. Therefore, it is difficult to identify them based on X-ray findings completely.

**FIGURE 1:**
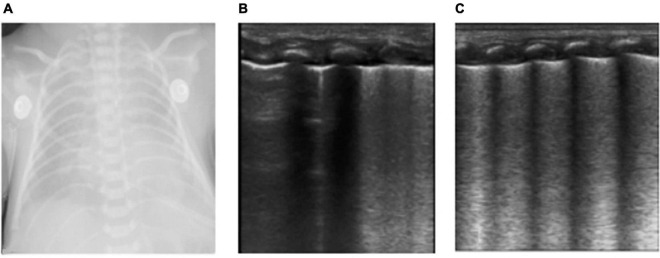
The neonate was born at 36 weeks of gestational age by Cesarean section due to the mother’s central placenta previa hemorrhage. The amniotic fluid was clear, and the Apgar score of 1, 5, and 10 min of birth were 10 points. The neonate had labored breathing and shortness of breath 10 min after delivery and mechanical ventilation was required. It was diagnosed as severe wet lung. **(A)** Chest X-ray: Lung permeability decreases, and the white lung changes. **(B,C)** Ultrasound findings: Rough pleural line, reduced A-line, alveolar interstitial syndrome (AIS), double lung points but no consolidation, which conforms to the ultrasonic characteristics of wet lung. After 24 h of treatment, the neonate’s breathing improved. LUS showed that B-lines was significantly reduced and the patient was successfully evacuated from the ventilator. The clinical outcome was consistent with the characteristics of wet lung.

**FIGURE 2:**
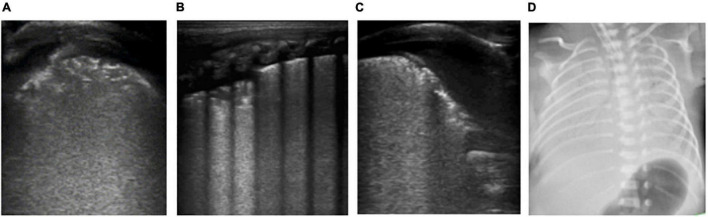
The patient is a twin neonate born at 32 weeks of gestational age by Cesarean section due to “fetal distress” with clear amniotic fluid and the Apgar score of 1, 5, and 10 min of birth were 5, 7, and 7 points. After delivery, the neonate had cyanosis, labored breathing, and severe pulmonary hypertension, and high-frequency ventilation was performed. It was diagnosed as neonatal respiratory distress syndrome (RDS). **(D)** Chest X-ray: White lung changes. **(A–C)** LUS: Abnormal pleural line, the disappearance of A-lines, white lung change, snowflake sign, such as air bronchogram visible below pleura, which conforms to the ultrasonic characteristics of severe RDS.

Lung ultrasound examination is mainly affected by the different proportion of air and fluid distribution in lung tissues; for example, normally inflated lung tissues (lung sliding, pleural line, and A-lines), pulmonary edema of varying degrees (B-lines, fusion of B-line, and compact B-lines), lung consolidation of varying degrees (air bronchogram), and other ultrasonic images. When LUS presents compact B-lines in each scanning area of both lungs, it indicates a white lung. Different from the “white lung” on X-ray, that on LUS indicates severe pulmonary edema. Ultrasound showed the disappearance of pleural line, A-lines, compact B-lines, and even white lung disappeared on both RDS and TTN. However, the ultrasonic diagnosis of TTN is based on varying degrees of pulmonary edema without lung consolidation (see Figures 1A–C), while the critical ultrasound imaging feature of RDS is lung consolidation accompanied by air bronchogram (see Figures 2A–D). The two can be distinguished according to the consolidation accompanied by air bronchogram (4). “Ground-glass opacity sign” is an early ultrasonic sign of RDS, which is characterized by strong near-field echo, gradually weakening from near-field to far-field echo, while line B has no attenuation. It can be used to distinguish RDS from TTN (5, 6). Table 1 summarizes the different characteristics of TTN and RDS ultrasound images.

**TABLE 1:** Differences of the ultrasonic images between TTN and RDS.

	TTN	RDS
Pleural line	Rough or vague	Discontinuous or disappeared
A-lines	Reduced or disappeared	Disappeared
B-lines	Fusion of B-line, compact B-lines, AIS, white lung	Fusion of B-line, compact B-lines, AIS, white lung
Double lung points	Mild TTN Recovery period of TTN	Acute stage of mild RDS Recovery period of severe RDS
Consolidation, air bronchogram	No such performance	Mild RDS: Ground-glass opacity sign Moderate and severe RDS: snowflake sign Severe cases may have atelectasis

## Rapid Identification of Urgent Respiratory Distress: Pleural Effusion, Pneumothorax, or Atelectasis?

**FIGURE 3:**
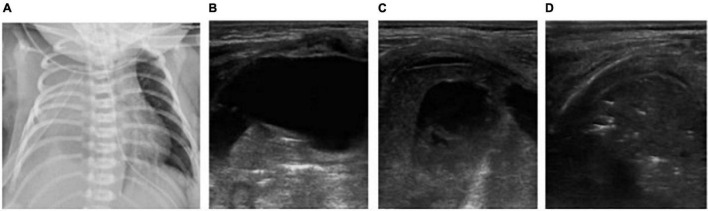
The patient was born at 36^+2^ weeks of gestational age and diagnosed as congenital esophageal atresia. After surgical treatment, man-machine incongruity appeared under mechanical ventilation. X-ray shows white lung on the right lung **(A)**. Atelectasis? or pleural effusion? X-ray failed to distinguish. But LUS clearly showed a large amount of pleural effusion on the right side, the right lung was compressed, showing a liver like echo and the strip bronchial inflation sign, suggesting atelectasis of the right lung **(B–D)**. After thoracic puncture and drainage and adjustment of thoracic drainage tube, the right atelectasis improved.

**FIGURE 4:**
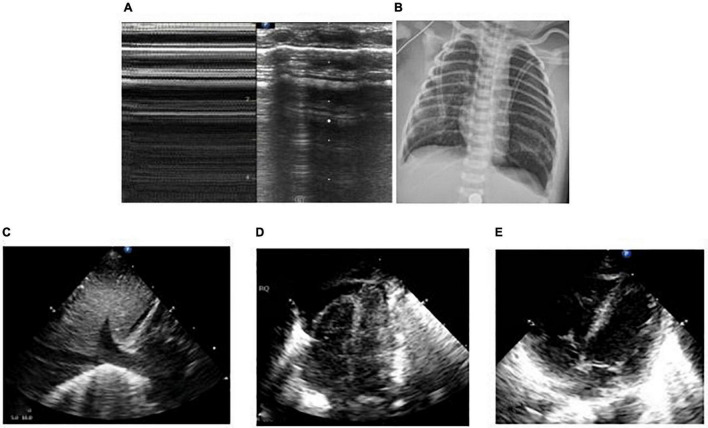
The patient was born at 39 weeks of gestational age by natural delivery. The amniotic fluid was III turbid, and the Apgar score of 1, 5, and 10 min of birth were 2, 3, and 6 points. Respiratory distress occurred after birth and requiring mechanical ventilation. It was diagnosed as neonatal meconium aspiration syndrome. Under mechanical ventilation, man-machine incongruity appeared. The patient had weakened breath sounds in both lungs, a heart rate of 120 beats/min, dull heart sound, pale skin tone, cool limb ends, and weak radial artery beats. The blood pressure was 56/35 mmHg, and the shock score was 5 points. Continuous hypoxemia appeared, the arterial blood gas was PO_2_ 30.4 mmHg, and calculated Oxygenation index (OI) value was 50. Emergency bedside ultrasound revealed the lung slip of both lungs disappeared and without B-line, stratospheric sign showed, and the lung point was not explored, suggesting the diagnosis of massive pneumothorax on both sides **(A)**. Emergency thoracocentesis was performed under ultrasonic monitoring. After the operation, the blood oxygen saturation increased, blood pressure rose to 65/40 mmHg and the heart sound was strong. After 30 min, the X-ray operator at the bedside arrived, and the X-ray chest film showed bilateral pneumothorax **(B)**. In emergency situations, bedside ultrasound is easier to obtain than X-ray examination. In the figure, who has massive pneumothorax on both sides, hours after thoracic puncture and drainage, the baby appeared to cyanosis, the breath sounds in both lungs was weak, the heart rate increased to 180 beats/min. Reexamination of LUS showed that pneumothorax decreased and the lower boundary of lung was too low, bedside cardiac ultrasound showed inferior vena cava expansion and fixation **(C)**, ventricular filling and relaxation limitation **(D)**, suggesting that the patient had excessive lung expansion. The ventilator pressure was reduced to increase the returning blood volume, and the heart rate decreased gradually to 150 beats/min. Ventricular filling and diastolic improvement were observed by ultrasound **(E)**.

The pulmonary lesions of critically ill patients are manifested as pulmonary consolidation, pulmonary edema, pleural effusion, and pneumothorax. As a non-invasive, real-time, and repeatable tool, LUS can achieve rapid bedside visualization of pulmonary diseases. Thus, it is a vital application in the field of critical ills (7). Sudden pneumothorax or massive pleural effusion causes atelectasis in a large area, affecting lung function. It is a common cause of sudden distress in neonates. Ultrasound is more accurate than decubitus abdominal radiograph and nearly as accurate as CT in detecting pleural effusion (8). Neonates are mostly in the supine position, and inflammation exudates deposit on the dorsal side under gravity. Therefore, pleural effusion is detected in the axillary and dorsal areas of both lungs, and atelectasis is detected in the dorsal and spinal areas of both lungs in most cases (see Figures 3A–D, 4A,B). Conversely, the primary purpose of examining the anterior sides of both lungs is to examine the pneumothorax.

## Guideding Lung Recruitment: Atelectasis or Overinflation?

**FIGURE 5:**
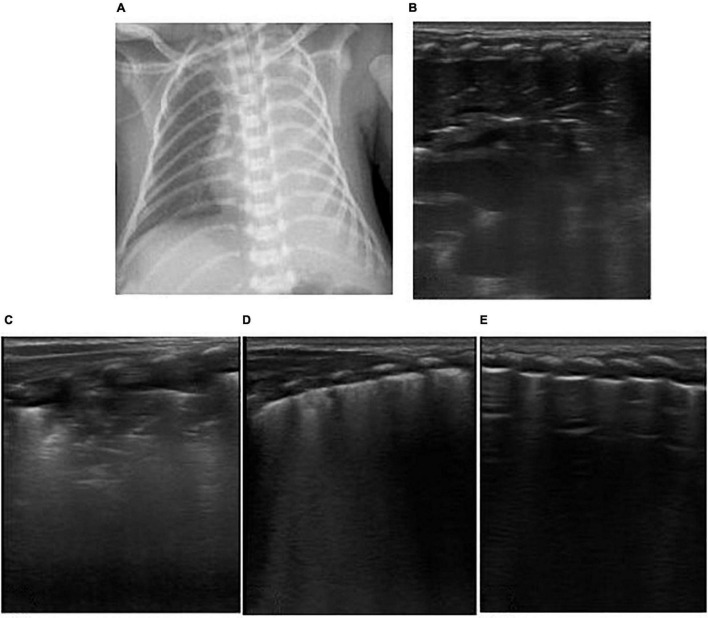
The patient was born at 33^+1^ weeks of gestational age by Cesarean section due to the mother’s placenta previa and hemorrhagic shock, with an inborn weight of 1.75 kg and hemorrhagic amniotic fluid. The Apgar score of 1, 5, and 10 min of birth were 1, 7, and 7 points. The patient had pulmonary hemorrhage after delivery. Ventilator weaning was performed after 10 days of mechanical ventilation. After 2 days post-ventilator weaning, the patient had decreased blood oxygen and labored breathing. Mechanical ventilation was reperformed *via* endotracheal intubation. Emergency LUS showed that the left lung showed liver like echo, pleural line, and A line disappeared, dendritic bronchial inflation sign and pulmonary pulsation were visible, revealed left lung atelectasis **(B)**. Chest X-ray showed white lung changes in the left lung **(A)**. Lung recruitment was performed under ultrasound monitoring, and the range of consolidation was reduced gradually, and pleural line and A-lines were clear **(C–E)**. Lung ventilation improved, and finally, the ventilator was weaned.

Atelectasis is a common complication of neonatal lung diseases, usually accompanied by NRDS, MAS, severe pneumonia, and other diseases. It is also the common cause of neonatal dyspnea. Neonates given mechanical ventilation often face man-machine incongruity or disengagement failure caused by atelectasis. Lung recruitment refers to a sustained increase in airway pressure or volume within a limited time to maximize the physiological expansion of the maximum lung units, with the goal to open the collapsed pulmonary alveoli, thus improving gas exchange, oxygenation function, and lung compliance. The ultrasound of neonatal atelectasis was characterized by hepatoid echo consolidation accompanied by lung pulsation and air bronchogram (9, 10), indicating serious alveolar collapse. With the increase in alveolar pressure during lung recruitment, alveolar inflation increased, and ventilation and blood flow ratio improved. Under LUS monitoring, dynamic air bronchogram increased, A-line gradually appeared, and B-line and consolidation decreased gradually. Moreover, changes in lung sliding were monitored, and positive pressure ventilation pressure was adjusted to avoid pneumothorax caused by alveolar overinflation (see Figures 4C–E, 5A–E).

Lung ultrasound can also be used to guide the precise care of patients with severe pneumonia. Since prolonged supine position leads to severe lung consolidation in gravity-dependent areas and insufficient ventilation due to dorsal alveoli closure, it is necessary to change position or perform prone ventilation to promote deep sputum drainage and reopen the dorsal alveoli.

## Monitoring Development of Pneumonia and Predicting Mechanical Ventilation

Currently, LUS has been widely studied with respect to mechanical ventilation in critically ill patients to guide weaning. However, only a few studies have reported the use of LUS in predicting the need for mechanical ventilation. The main ultrasound characteristics of pneumonia are abnormal pleural line, lung consolidation, and alveolar interstitial syndrome, and severe lung consolidation can be developed into atelectasis (11). The more serious the alveolar collapse, the more serious the lung consolidation, and the worse the lung function. In clinical practice, hepatoid consolidation is observed by ultrasound in the lungs of neonates with severe diseases or when lung consolidation occurs in non-gravity-dependent areas, the range of lung consolidation in gravity-dependent areas has been very extensive and serious, necessitating mechanical ventilation for treatment. Therefore, for newly hospitalized neonates with dyspnea as the chief complaint, LUS should be performed as soon as possible. When lung consolidation with hepatoid echo or in non-gravity-dependent areas occurs, respiratory support should be provided at the earliest. In severe cases, invasive mechanical ventilation should be performed.

**FIGURE 6:**
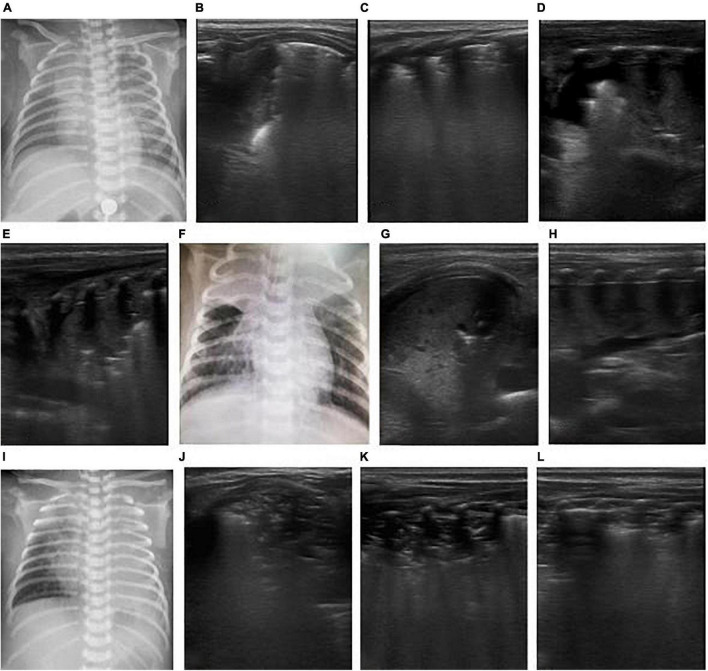
The patient was a 26-day-old, full-term neonate and admitted to the hospital due to cough and shortness of breath for 4 days and aggravation for 2 days. The neonate had a spasmodic cough, and his mother had a history of cough. The nucleic acid test of respiratory virus suggested pertussis. It was diagnosed as severe pertussis pneumonia. On the admission day, LUS showed bilateral consolidation with atelectasis in the right upper lung **(B,C)**, and chest X-ray showed consolidation of the right upper lung **(A)**. After admission, the patient was given anti-infection and CPAP respiratory support, but after half a day, the respiratory distress progressively worsened, and blood oxygen decreased. Re-examination by LUS showed increased consolidation of both lungs as a hepatoid echo and developed to a large area atelectasis. Bronchial inflation sign was reduced, and consolidation was severe **(D–F)**. Hence, mechanical ventilation was provided *via* endotracheal intubation for respiratory support. An emergency exchange transfusion was performed. WBC increased again after 3 days of hospitalization, and atelectasis was aggravated **(G–I)**, and exchange transfusion was performed again. After the second exchange transfusion, ultrasound monitored pulmonary atelectasis was improved **(J–L)**.

Patients with severe pertussis are prone to pulmonary consolidation and atelectasis (12–14). When LUS shows no lung consolidation, the patients usually do not need invasive mechanical ventilation, or only need non-invasive (CPAP, HFNC) respiratory support. When LUS indicates different degrees of lung consolidation, or consolidation progresses to atelectasis, it indicates the need for invasive mechanical ventilation (see Figures 6A–L).

## Discussion

**FIGURE 7:**
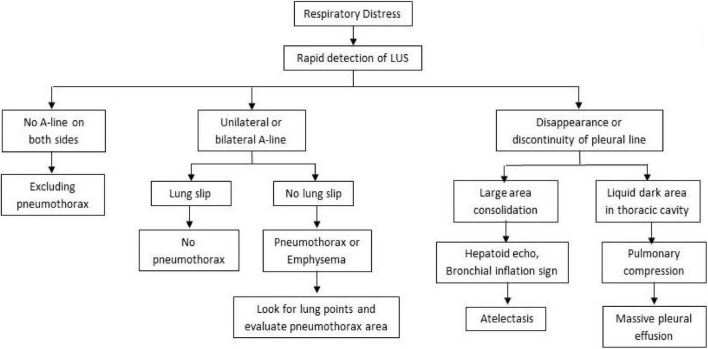
Process of rapid detection of respiratory distress by LUS.

Summarizing the above cases, we found that in the treatment of neonatal respiratory critical illness, the application of LUS to monitor pulmonary lesions is more advantageous than X-ray. Both atelectasis and a large amount of pleural effusion show decreased transparency or white lung on X-ray chest film, which is difficult to distinguish, but the ultrasonic manifestations of the two have different signs, which are easy to distinguish. The accuracy and sensitivity of LUS diagnosis of pneumothorax are higher than that of X-ray, especially a small amount of pneumothorax. The X-ray manifestations of TTN and RDS are similar, while ultrasound can distinguish them according to the presence of consolidation and bronchial inflation sign (see Figure 7). In addition, ultrasound has no radiation damage, and can be carried out at the bedside as soon as possible in case of emergency, which is timelier and safer than X-ray.

Children’s lung ultrasound technology in our country began in 2010. Professor Liu Jing, an expert in pediatric LUS, propagated its application in mature ways in China (15). The consensus on LUS guidelines for neonatal RDS and pneumothorax has been established in recent years (5, 16). Some studies have differentiated the mild to moderate severity of RDS and pneumothorax and pointed out thoracentesis is safe under ultrasonic positioning, rendering it unnecessary to limit it to traditional position and puncture site. These features indicated that LUS is no longer limited to qualitative assessment of pathological changes in the lung. Semiquantitative assessment and functional application could fulfill the needs of clinical diagnosis and treatment. Hitherto, some studies assessed the quantitative schemes of LUS: for example, De Martino et al. (17) pointed out that semiquantitative pulmonary ultrasound score was used to predict the need for surfactant dose in extremely premature neonates; Jing et al. (18) utilized an animal model and showed that varied pulmonary water content has corresponding manifestations in ultrasound and is related to different degrees of clinical symptoms. LUS can roughly estimate the extravascular pulmonary water content according to B-line type. In the diagnosis and treatment of infectious pneumonia, Jing et al. (19) showed that LUS detects lung consolidation earlier than the increase of infection indicators, such as CRP and PCT, confirming that LUS was highly sensitive in diagnosing pneumonia. However, the etiological examination to identify the pathogen causing the pneumonia is essential, and LUS has no specific images. The characteristics of LUS images of pneumonia with different etiologies are under exploration. We found that the lung consolidation of neonates with community-acquired pneumonia was mostly located in the right upper dorsal lung and was most serious in pertussis pneumonia. Thus, the precise treatment and nursing of patients with pneumonia is crucial.

## Conclusion

Lung ultrasound is a rapid, accurate, and non-invasive tool for the diagnosis of neonatal respiratory critical diseases. It avoids radiation exposure, is easy for repetitive bedside operation, and has high sensitivity in the diagnosis of pulmonary edema, pneumothorax, lung consolidation, and atelectasis, making up for the deficiency of X-ray. When LUS examination is difficult in subcutaneous emphysema and deep lesions, X-ray and LUS can complement each other, improving the accuracy of diagnosis (20). LUS is one of the major technologies in the detection of lung diseases. It is recommended that medical staff in ICUs become proficient and promote LUS.

## Data Availability Statement

The original contributions presented in the study are included in the article/supplementary material, further inquiries can be directed to the corresponding author/s.

## Ethics Statement

The studies involving human participants were reviewed and approved by the Ethics Committee of Dongguan Eighth People’s Hospital. Written informed consent to participate in this study was provided by the participants’ legal guardian/next of kin. Written informed consent was obtained from the individual(s), and minor(s)’ legal guardian/next of kin, for the publication of any potentially identifiable images or data included in this article.

## Author Contributions

BD conceptualized the study, collected and analyzed the data, and wrote the manuscript. FX assisted with the study design and reviewed the drafts of the manuscript. JLi, MM, QC, and JLiao assisted with the data collection and analysis. XH and NL conceptualized the study and reviewed the drafts of the manuscript. All authors contributed to the article and approved the submitted version.

## Conflict of Interest

The authors declare that the research was conducted in the absence of any commercial or financial relationships that could be construed as a potential conflict of interest.

## Publisher’s Note

All claims expressed in this article are solely those of the authors and do not necessarily represent those of their affiliated organizations, or those of the publisher, the editors and the reviewers. Any product that may be evaluated in this article, or claim that may be made by its manufacturer, is not guaranteed or endorsed by the publisher.
